# Metabolic Profiling of the *Uncaria Hook* Alkaloid Geissoschizine Methyl Ether in Rat and Human Liver Microsomes Using High-Performance Liquid Chromatography with Tandem Mass Spectrometry

**DOI:** 10.3390/molecules20022100

**Published:** 2015-01-27

**Authors:** Hirotaka Kushida, Takashi Matsumoto, Yasushi Igarashi, Hiroaki Nishimura, Junko Watanabe, Kazuya Maemura, Yoshio Kase

**Affiliations:** 1 Tsumura Research Laboratories, Tsumura & Co., 3586 Yoshiwara, Ami-machi, Inashiki-gun, Ibaraki 300-1192, Japan; E-Mails: matsumoto_takashi@mail.tsumura.co.jp (T.M.); watanabe_junko@mail.tsumura.co.jp (J.W.); maemura_kazuya@mail.tsumura.co.jp (K.M.); kase_yoshio@mail.tsumura.co.jp (Y.K.); 2 Kampo Formulation Development Center, Tsumura & Co., 3586 Yoshiwara, Ami-machi, Inashiki-gun, Ibaraki 300-1192, Japan; E-Mails: igarashi_yasushi@mail.tsumura.co.jp (Y.I.); nishimura_hiroaki@mail.tsumura.co.jp (H.N.)

**Keywords:** geissoschizine methyl ether, *Uncaria hook*, metabolic profile, LC/MS/MS, rat liver microsome, human liver microsome

## Abstract

Geissoschizine methyl ether (GM) is an indole alkaloid found in *Uncaria hook*, which is a galenical constituent of yokukansan, a traditional Japanese medicine. GM has been identified as the active component responsible for anti-aggressive effects. In this study, the metabolic profiling of GM in rat and human liver microsomes was investigated. Thirteen metabolites of GM were elucidated and identified using a high-performance liquid chromatography with tandem mass spectrometry method, and their molecular structures were proposed on the basis of the characteristics of their precursor ions, product ions, and chromatographic retention times. There were no differences in the metabolites between the rat and human liver microsomes. Among the 13 identified metabolites, there were two demethylation metabolites, one dehydrogenation metabolite, three methylation metabolites, three oxidation metabolites, two water-adduct metabolites, one di-demethylation metabolite, and one water-adduct metabolite followed by oxidation. The metabolic pathways of GM were proposed on the basis of this study. This study will be helpful in understanding the metabolic routes of GM and related *Uncaria hook* alkaloids, and provide useful information on the pharmacokinetics and pharmacodynamics. This is the first report that describes the separation and identification of GM metabolites in rat and human liver microsomes.

## 1. Introduction

**Figure 1:**
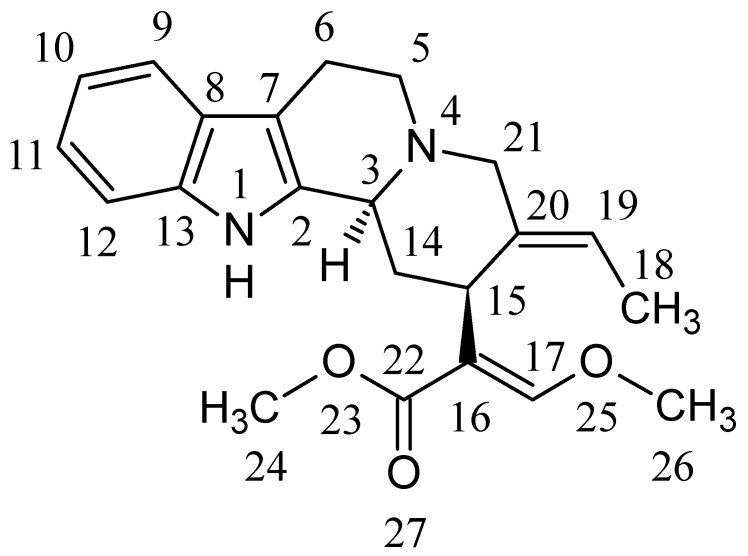
Chemical structure of GM.

Geissoschizine methyl ether [(16*E*,19*E*)-16,17,19,20-tetradehydro-17-methoxycorynan-16-carboxylic acid methyl ester, GM, Figure 1] is a major indole alkaloid found in *Uncaria hook*, which is a galenical constituent of yokukansan, a traditional Japanese medicine (Kampo). GM is considered to be one of the active components that contribute to the psychotropic effect of yokukansan [1,2,3,4]. For example, GM has a partial agonist effect on serotonin (5-HT)_1A_ receptors, which mediates amelioration of increased aggressiveness and decreased sociality in isolation-stressed mice [4]. Recently, we demonstrated that GM had an antagonistic effect on the 5-HT_7_ receptor [5]. Ueda *et al.* also reported that GM behaved as a partial agonist at 5-HT_1A_ receptors, a partial agonist/antagonist at the dopamine D_2L_ receptor, andan antagonist at 5-HT_2A_, 5-HT_2C_, and 5-HT_7_ receptors [6]. Pharmacokinetic studies of the *Uncaria hook* alkaloids corynoxeine, isocorynoxeine, rhynchophylline, isorhynchophylline, hirsutine, hirsuteine, and GM after the oral administration of yokukansan in rats have also been conducted [7]. In the plasma, rhynchophylline, hirsutine, hirsuteine, and GM were detected; however, only GM was detected in the brain. These results suggest that GM is an active component contributing to the pharmacological effect of yokukansan.

In general, Kampo is orally administered, and the components in the extract are metabolized by gut flora and various enzymes. Hence, to evaluate the activity of Kampo, investigation of the metabolic fate of the constituents is very important. For example, the metabolic fate of hirsutine and hirsuteine, indole alkaloids with chemical structures similar to that of GM, was investigated in rats [8]. The metabolites of hirsutine and hirsuteine were isolated and identified as 11-hydroxyhirsutine and 11-hydroxyhirsuteine. The glucuronides of two 11-hydroxyindoles were also isolated and identified [8]. Furthermore, Le Verge *et al.* reported that yohimbine, which contains a tetracyclic indole alkaloid skeleton similar to that of GM, is metabolized to 10- and 11-monohydroxy forms in humans [9]. The compound 11-hydroxyyohimbine possesses the same α-2-adrenoceptor blocking activity as yohimbine [10]. More recently, the metabolism of yokukansan in rat urine was investigated, and 18-hydroxy GM was identified as the metabolite of GM [11]. However, the other metabolites of GM except for 18-hydroxy GM were not detected in rat urine. The metabolic profile of GM has not yet been sufficiently investigated. Therefore, to examine the activity of yokukansan, the metabolic fate of GM must be investigated.

Nuclear magnetic resonance (NMR) techniques are frequently used to determine the structures of drug metabolites. A large amount of refined metabolites is necessary for structure determination by NMR. However, the separation and refinement of metabolites are difficult when many metabolites are produced. Recently, high-performance liquid chromatography with tandem mass spectrometry (LC/MS/MS) became a powerful and frequently used technique for metabolite separation and identification. The identification of drug metabolites by LC/MS/MS techniques involves two major steps: detection of drug metabolite precursor ions and acquisition of their MS^2^ spectra for structural characterization [12]. LC/MS/MS techniques were very useful for the identification of metabolites in the preliminary study. Furthermore, hirsutine and hirsuteine are metabolized by cytochrome P450s (CYPs) in rat liver microsomes [8]; thus, it is predicted that GM is also metabolized by enzymes (e.g., CYPs) in liver microsomes. In addition, it is important to examine the differences in the metabolites produced between experimental animal (e.g., rat) and human. Therefore, in this study, to evaluate the metabolic profile of GM, the metabolites of GM obtained from rat and human liver microsomes were investigated using LC/MS/MS, and the MS^2^ spectra were used to determine their composition and structure.

## 2. Results and Discussion

### 2.1. Identification of GM Metabolites Using LC/MS/MS

A pharmacokinetic study using the LC/MS/MS method for *Uncaria hook* alkaloids, including GM, has previously been reported by our laboratory [7], and this approach was found to be feasible and sensitive. The same compositions of the mobile phase (gradient elution with 0.2% formic acid and acetonitrile) were used in this study for qualitative analysis of GM and its metabolites.

**Figure 2:**
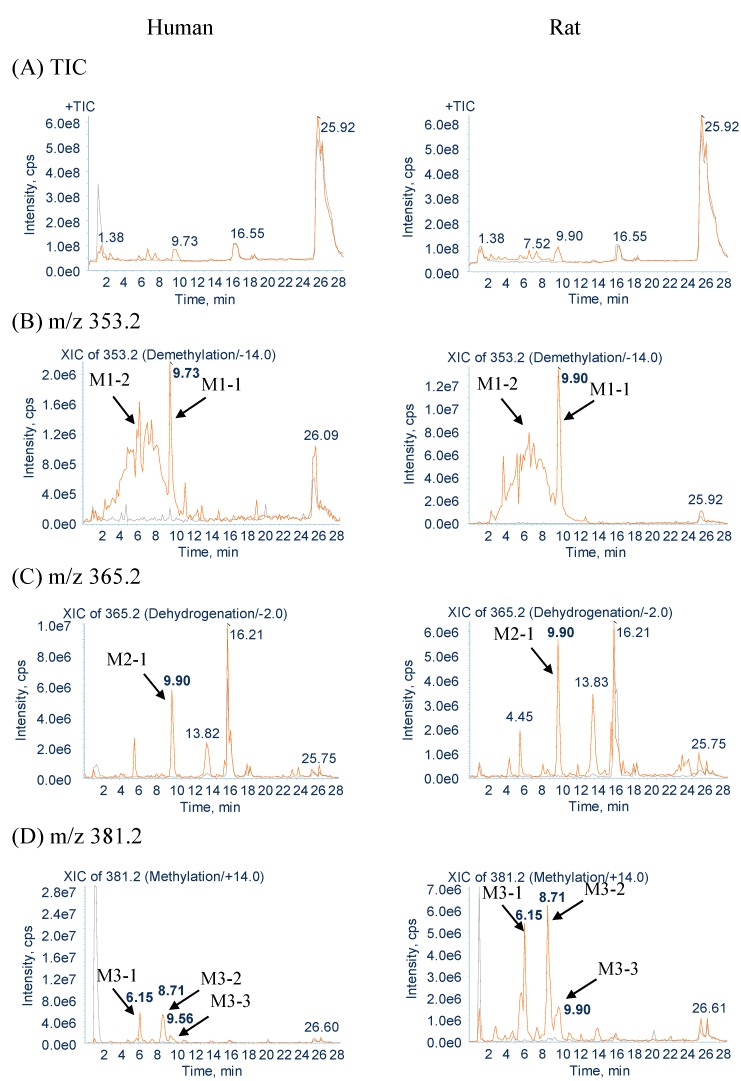

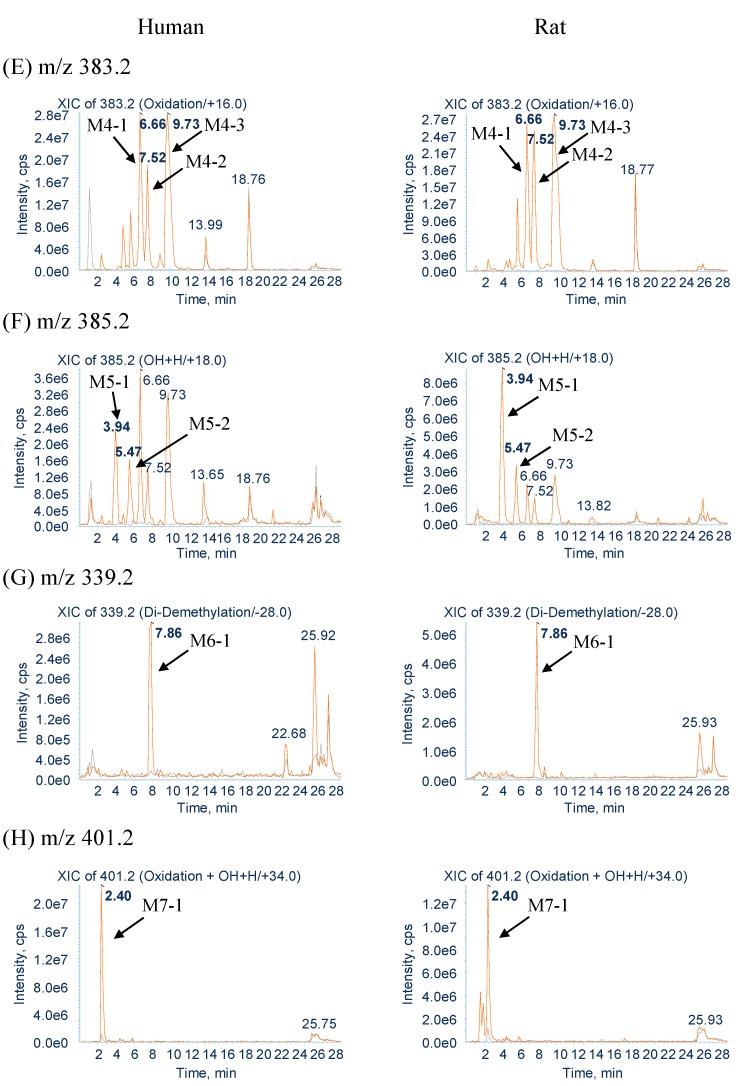
Full-scan total-ion and extracted-ion chromatograms of GM metabolites in rat and human microsomes. (**A**) Full-scan total-ion chromatogram (*m*/*z* 10–500); (**B**) demethylation metabolites (*m*/*z* 353.2); (**C**) dehydrogenation metabolites (*m*/*z* 365.2); (**D**) methylation metabolites (*m*/*z* 381.2); (**E**) oxidation metabolites (*m*/*z* 383.2); (**F**) water-adduct metabolites (*m*/*z* 385.2); (**G**) di-demethylation metabolites (*m*/*z* 339.2); (**H**) water-adduct metabolite followed by oxidation (*m*/*z* 401.2).

The samples were analyzed in positive-ion modes to identify GM and its metabolites. Figure 2 shows the total-ion and extracted-ion chromatograms of GM and its metabolites after 60 min of incubation with rat and human liver microsomes. Compared with the peaks in the blank sample, the test samples contained at least 13 additional peaks (assumed to be metabolites). There were no differences in the metabolites produced between the rat and human liver microsomes. This result suggests that the same *in vivo* metabolites are biotransformed in rats and humans. There was no difference in metabolite production between the species. Production of these metabolites may be mainly classified into five routes: demethylation, dehydrogenation, methylation, oxidation, and water-adduct metabolism. The 13 identified metabolites included M1-1 and M1-2 from demethylation (−14 from the parent), M2-1 from dehydrogenation (−2 from the parent), M3-1–M3-3 from methylation (+14 from the parent), M4-1–M4-3 from oxidation (+16 from the parent), M5-1 and M5-2 from the water adduct (+18 from the parent), M6-1 (*m*/*z* 339.2) from di-demethylation (−24 from the parent), and M7-1 from the water adduct followed by oxidation (+34 from the parent). These metabolites were numbered according to their *m*/*z* in the extracted-ion chromatograms, number of metabolism, and retention time.

Nakazawa *et al.* reported that the metabolites of hirsutine and hirsuteine, the indole alkaloids with a chemical structure similar to that of GM, were identified as 11-hydroxyhirsutine and 11-hydroxyhirsuteine in rats [8]. However, the other metabolites, except for the oxidation metabolites, were not identified. In this study, five metabolic routes, demethylation, dehydrogenation, methylation, oxidation, and water adduct, were confirmed in the metabolism of GM. This result suggests that the indole alkaloids, hirsutine, hirsuteine, and GM in *Uncaria hook* may be metabolized through various metabolic routes.

### 2.2. Structure Elucidation of GM Metabolites in Rat and Human Liver Microsomes

#### 2.2.1. Metabolites Produced by Demethylation (M1-1 and M1-2)

**Figure 3:**
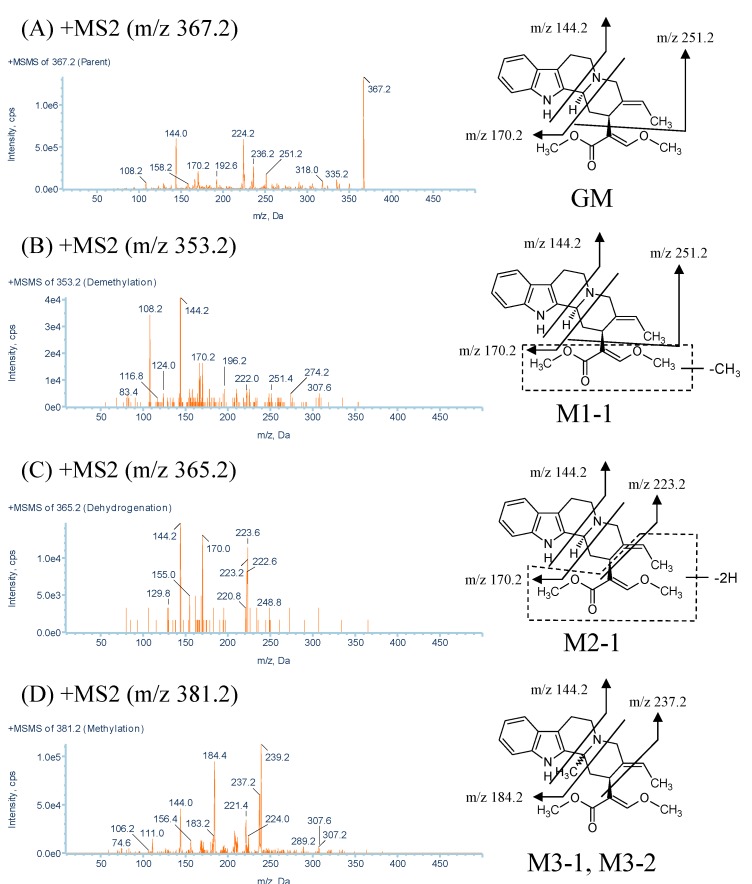

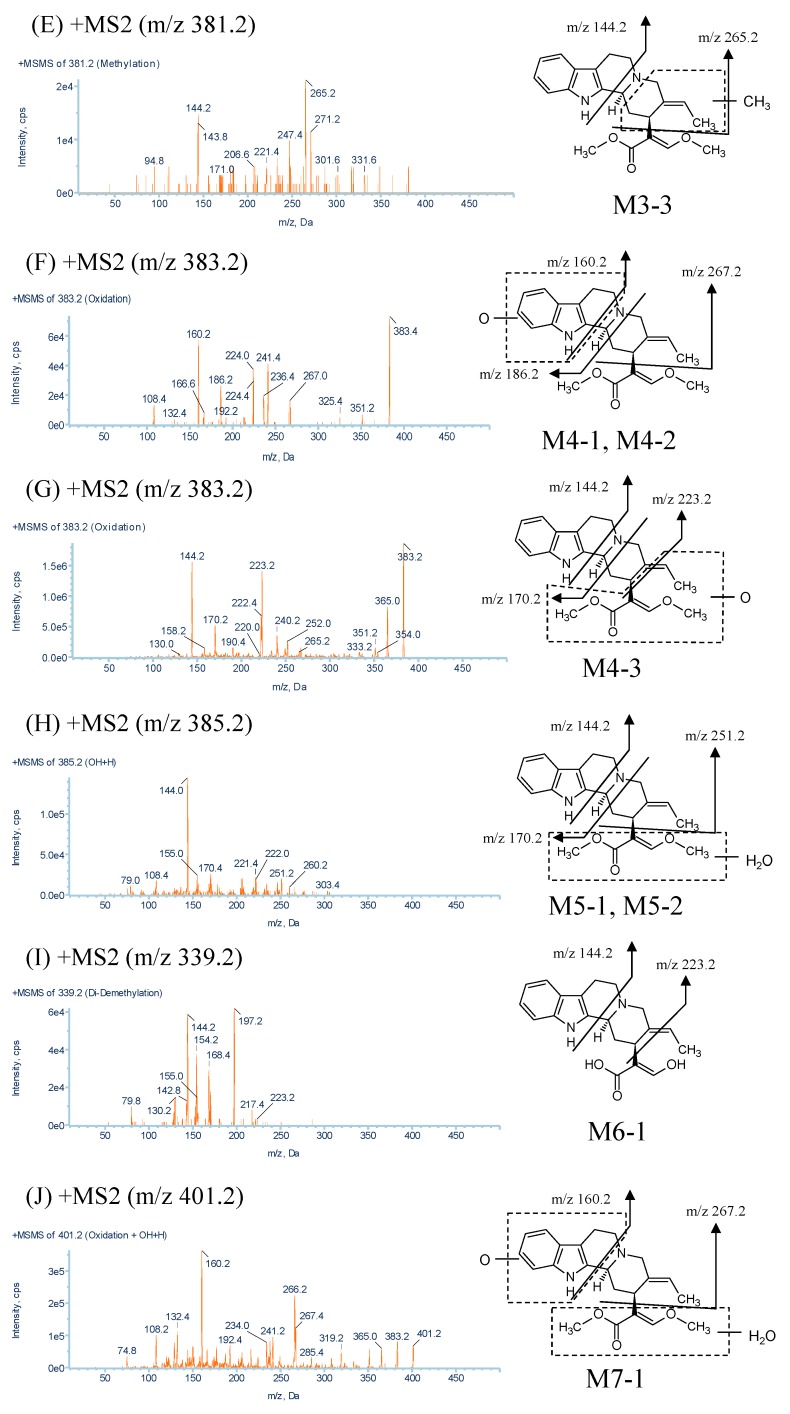
MS^2^ spectra of GM and its metabolites. (**A**) GM; (**B**) methylation metabolite (M1-1); (**C**) dehydrogenation metabolite (M2-1); (**D**) methylation metabolites (M3-1 and M3-2); (**E**) methylation metabolite (M3-3); (**F**) oxidation metabolites (M4-1 and M4-2); (**G**) oxidation metabolite (M4-3); (**H**) water-adduct metabolites (M5-1 and M5-2); (**I**) di-demethylation metabolite (M6-1); and (**J**) water-adduct metabolite followed by oxidation (M7-1) of GM.

In the rat and human liver microsomal incubations, M1-1 was detected at approximately 9.7 min, with a protonated molecular weight of 353.2 (*m*/*z* 367.2 − 14), which indicates the loss of one methyl group from the molecule (Figure 2). The MS^2^ spectra of M1-1 showed a series of the same characteristic fragment ions as those of GM at *m*/*z* 251.2, 170.2, and 144.2 (Figure 3A,B and Table 1). It was predicted that the 23-*O*- or 25-*O*-methyl group of GM was demethylated. Therefore, to identify metabolite M1-1, we synthesized 23-*O*-demethyl GM and 25-*O*-demethyl GM [(±)-geissoschizine] and compared the extracted-ion LC/MS/MS chromatograms to identify the structure of M1-1.

**Table 1:** Masses, retention times, and formulae of GM and its metabolites in rat and human liver microsomes, as detected by LC/MS/MS with positive electrospray ionization.

No.	Parent Ion *m*/*z*	Retention Time (min)	Formula	Product Ions (MS^2^)
GM	367.2	16.5	C_22_H_26_N_2_O_3_	335.2, 251.2, 236.2, 170.2, 144.0
M1-1	353.2	9.7	C_21_H_24_N_2_O_3_	251.4, 170.2, 144.2, 108.2
M2-1	365.2	9.9	C_22_H_24_N_2_O_3_	223.2, 170.0, 144.2
M3-1, M3-2, M3-3	381.2	6.1, 8.7, 9.5	C_23_H_28_N_2_O_3_	237.2, 184.4, 144.0, 265.2, 144.2
M4-1, M4-2, M4-3	383.2	6.6, 7.5, 9.7	C_22_H_26_N_2_O_4_	267.0, 186.2, 160.2, 223.2, 170.2, 144.2
M5-1, M5-2	385.2	3.9, 5.4	C_22_H_28_N_2_O_4_	251.2, 170.4, 144.0
M6-1	339.2	7.8	C_20_H_22_N_2_O_3_	223.2, 197.2, 168.4, 154.2, 144.2
M7-1	401.2	2.4	C_22_H_28_N_2_O_5_	267.4, 160.2

**Figure 4:**
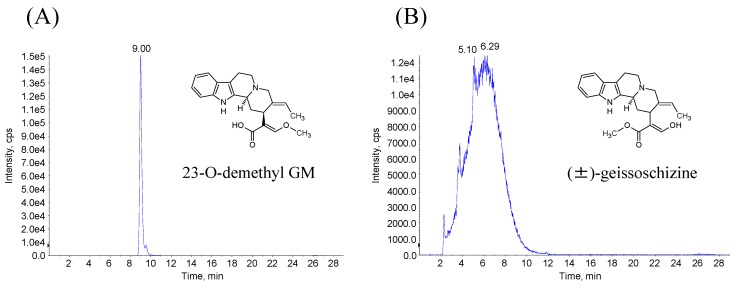
Demethylation metabolites of GM (M1-1 and M1-2). Extracted-ion chromatograms of 23-*O*-demethyl GM (**A**) and (±)-geissoschizine (**B**).

Figure 4 shows the extracted-ion chromatograms of 23-*O*-demethyl GM and (±)-geissoschizine. The peak of 23-*O*-demethyl GM with an *m*/*z* of 353.2 was detected at approximately 9.0 min, which matched the retention time of M1-1 in the microsomal incubations (Figure 4A). Furthermore, the peak of (±)-geissoschizine was broad and matched that before M1-1 in the chromatograms of the microsomal incubations (Figure 4B). The demethylation metabolites with broad peaks were numbered M1-2. These results suggest that GM was metabolized to two demethylation metabolites in rat and human liver microsomes.

#### 2.2.2. Metabolites Produced by Dehydrogenation (M2-1)

M2-1 was detected at approximately 9.9 min, with a protonated molecular weight of 365.2 (*m*/*z* 367.2 − 2), which was 2 Da lower than that of the protonated parent drug and indicated dehydrogenation containing the formation of a C–C bond (Figure 2). The MS^2^ spectra showed fragment ions at *m*/*z* 223.2, 170.2, and 144.2 (Figure 3C and Table 1). The fragment ions of *m*/*z* 170.2 and 144.2 were the same as those observed between M2-1 and GM, and the fragment ion of *m*/*z* 223.2 indicated that the −2 modification occurred on the side-chain moiety. However, the position of the dehydrogenation was unclear.

Therefore, M2-1 was predicted to be a dehydrogenation metabolite occurring at the side-chain moiety. Further characterization of the metabolite by NMR would be needed to fully characterize its structures in the future.

#### 2.2.3. Metabolites Produced by Methylation (M3-1, M3-2 and M3-3)

M3-1, M3-2 and M3-3 were detected at approximately 6.1, 8.7, and 9.5 min, respectively, each with a protonated molecular weight of 381.2 (*m*/*z* 367.2 + 14), which was 14 Da higher than that of the protonated parent drug and indicated methylation (Figure 2). The MS^2^ spectra of M3-1 and M3-2 showed fragment ions at *m*/*z* 237.2 (223.2→237.2), 184.2 (170.2→184.2), and 144.2 (Figure 3D and Table 1). The fragment ions of *m*/*z* 237.2 and 184.2 indicated that the +14 modification occurred on the moiety of tryptoline(1,2,3,4-tetrahydro-9*H*-pyrido[3,4,β]indole), and the absence of the fragment ion of *m*/*z* 144.2 indicated that methylation occurred at C-3. The MS^2^ spectra of the two metabolites showed exactly the same fragmentation pattern, suggesting that the two peaks were isomers. We hypothesize that M3-1 and M3-2 were isomers produced by methylation at C-3. The MS^2^ spectra of M3-3 showed fragment ions at *m*/*z* 265.2 (251.2→265.2) and 144.2 (Figure 3E and Table 1). The fragment ions of *m*/*z* 237.2 and 184.2 were not detected but that of *m*/*z* 265.2 was detected. These results suggest that the region surrounded by the dot-line frame shown in Figure 3E was methylated. However, the position of the methylation was unclear. Therefore, M3-3 was predicted to be a methylation metabolite occurring at the region surrounded by the dot-line frame. Further characterization of the metabolite by NMR would be needed to fully characterize its structures in the future.

#### 2.2.4. Metabolites Produced by Oxidation (M4-1, M4-2 and M4-3)

M4-1, M4-2 and M4-3 were detected at approximately 6.6, 7.5, and 9.7 min, respectively, each with a protonated molecular weight of 383.2 (*m*/*z* 367.2 + 16), which was 16 Da higher than that of the protonated parent drug and indicated oxidation (Figure 2). The MS^2^ spectra of M4-1 and M4-2 showed fragment ions at *m*/*z* 267.2 (251.2→267.2), 186.2 (170.2→186.2), and 160.2 (144.2→160.2) (Figure 3F and Table 1). The fragment ion of *m*/*z* 160.2 was attributed to the oxidation of the region surrounded by the dot-line frame shown in Figure 3F, which suggested that the +16 modification occurred on the region surrounded by the dot-line frame. However, the position of the oxidation was unclear. Therefore, M4-1 and M4-2 were predicted to be oxidation metabolites occurring at the region surrounded by the dot-line frame. The MS^2^ spectra of M4-3 showed fragment ions at *m*/*z* 223.2, 170.2, and 144.2 (Figure 3G and Table 1). The fragment ions of *m*/*z* 170.2 and 144.2 were the same as those observed between M4-3 and GM, and the fragment ion of *m*/*z* 223.2 indicated that the +16 modification occurred on the side-chain moiety. However, the position of the oxidation on these metabolites was unclear. Therefore, M4-3 was predicted to be an oxidation metabolite occurring at the side-chain moiety. Further characterization of the metabolite by NMR would be needed to fully characterize its structures in the future.

The metabolic fate of hirsutine and hirsuteine, the indole alkaloids with a chemical structure similar to GM, was investigated in rats [8]. The metabolites of hirsutine and hirsuteine were isolated and identified as 11-hydroxyhirsutine and 11-hydroxyhirsuteine. Le Verge *et al.* also reported that yohimbine, which contains a tetracyclic indole alkaloid skeleton similar to those of GM, is metabolized to 10- and 11-monohydroxy forms in humans [9]. In this study, the oxidation metabolites (M4-1 and M4-2), hydroxy form, at the indole skeleton of GM were confirmed. M4-1 and M4-2 might also have 10- and 11-mono-hydroxy forms. More recently, 18-hydroxy GM was identified as the metabolite of GM in rat urine administrated with yokukansan [11]. M4-3 is predicted to be an oxidation metabolite at the side chain moiety containing C-18. M4-3 might be 18-hydroxy GM. Furthermore, it was reported that the glucuronide of 11-hydroxy hirsutine and hirsuteine was identified [8]. In the preliminary study, the glucuronic acid conjugates of hydroxy GM were predicted to be in rat bile samples. The glucuronic acid conjugation of hydroxy GM might also occur *in vivo*. Further identification of the phase II metabolites by an *in vivo* study would be required to fully understand the metabolic fate of GM in the future.

#### 2.2.5. Metabolites Produced by Water-Adduct Metabolism (M5-1 and M5-2)

M5-1 and M5-2 were detected at approximately 3.9 and 5.4 min, respectively, each with a protonated molecular weight of 385.2 (*m*/*z* 367.2 + 18), which was 18 Da higher than that of the protonated parent drug and indicated water addition (Figure 2). The MS^2^ spectra of M5-1 and M5-2 showed fragment ions at *m*/*z* 251.2, 170.2, and 144.2 (Figure 3H and Table 1). The fragment ions of *m*/*z* 170.2 and 144.2 were the same as those observed between the water-adduct metabolites and GM, and the fragment ion of *m*/*z* 251.2 indicated that the +18 modification occurred on the side-chain moiety. However, the position of the water addition was unclear. Therefore, M5-1 and M5-2 were predicted to be water-adduct metabolites occurring at the side chain moiety. Further characterization of the metabolite by NMR would be needed to fully characterize its structures in the future.

#### 2.2.6. Metabolites Produced by Di-Demethylation (M6-1)

M6-1 was detected at approximately 7.8 min and had a protonated molecular weight of 339.2 (*m*/*z* 367.2 − 28), which was 28 Da lower than that of the protonated parent drug and indicated di-demethylation (Figure 2). The MS^2^ spectra showed fragment ions at *m*/*z* 223.2 and 144.2 (Figure 3I and Table 1). The fragment ion of *m*/*z* 144.2 was the same as that observed between M6-1 and GM, and the fragment ion of *m*/*z* 223.2 indicated that the −28 modification occurred on the side-chain moiety. In this study, we demonstrated that the 23-*O*- and 25-*O*-methyl group of GM was demethylated. Therefore, M6-1 was predicted to be a di-demethylation metabolite of GM.

#### 2.2.7. Metabolites Produced by Oxidation Followed by Water Addition (M7-1)

M7-1 was detected at approximately 2.4 min, with a protonated molecular weight of 401.2 (*m*/*z* 367.2 + 34), which was 34 Da higher than that of the protonated parent drug and indicated the introduction of an oxygen atom with water addition (Figure 2) The MS^2^ spectra of M7-1 showed fragment ions at *m*/*z* 267.2 (251.2→267.2) and 160.2 (144.2→160.2) (Figure 3J and Table 1). The fragment ion of *m*/*z* 160.2 indicated that oxidation occurred on the region surrounded by the dot-line frame, and that of *m*/*z* 267.2 indicated that water addition occurred on the side-chain moiety (Figure 3J). These results suggest that the +16 and +18 modifications occurred on the region surrounded by the dot-line frame and side-chain moiety. However, the positions of oxygen and water addition on this metabolite were unclear. Therefore, M7-1 was predicted to be an oxidation metabolite followed by water-addition at the side chain moiety.

### 2.3. Biotransformation Pathway of GM

**Scheme 1:**
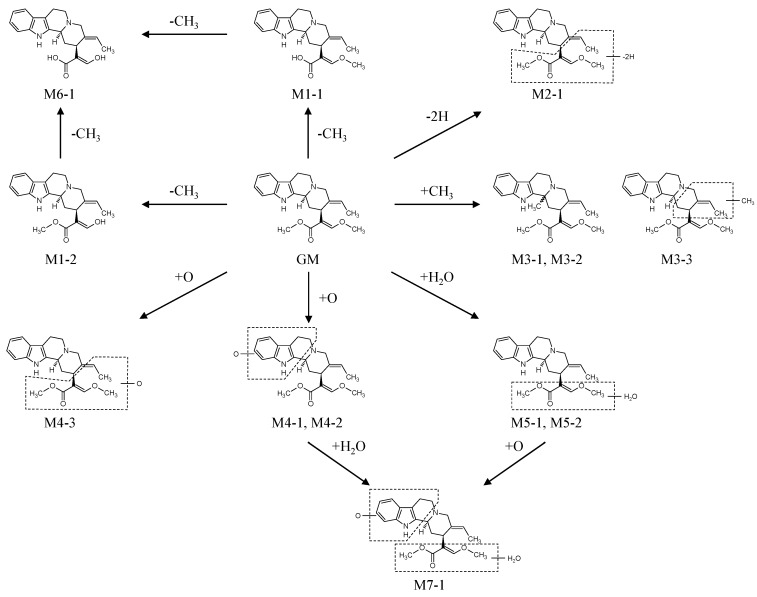
Proposed *in vitro* metabolic pathways of GM in rat and human microsomes.

According to the chemical structures of GM and its metabolites, the possible metabolic pathways illustrated in Scheme 1 are proposed and include demethylation metabolites (M1-1 and M1-2), a dehydrogenation metabolite (M2-1), methylation metabolites (M3-1–M3-3), oxidation metabolites (M4-1–M4-3), water-adduct metabolites (M5-1 and M5-2), a di-demethylation metabolite (M6-1), and a water-adduct metabolite followed by oxidation (M7-1).

## 3. Experimental Section

### 3.1. Chemicals and Reagents

#### 3.1.1. Isolation of GM

Figure 1 shows the chemical structure of GM. GM was isolated from *Uncaria hook* in Tsumura & Co. (Ibaraki, Japan), according to the previously described method [13].

#### 3.1.2. Synthesis of 23-*O*-demethyl GM and (±)-Geissoschizine

23-*O*-Demethyl GM and 25-*O*-demethyl GM [(±)-geissoschizine] were synthesized by Tsumura & Co. In brief, GM was dissolved in 1,4-dioxane and hydrolyzed under alkaline conditions by lithium hydroxide for 2 h at 50 °C to produce 23-*O*-demethyl GM, in which the methylester in the GM structure was converted to carbonic acid (refer to Figure 1). The reactant solution was neutralized by adding HCl and then filtered through a Sephadex LH-20 column (GE Healthcare, Buckinghamshire, UK) to remove inorganic salt. The resultant solution was evaporated to dryness, and solid 23-*O*-demethyl GM was subsequently obtained. (±)-Geissoschizine was synthesized from tryptamine by the modification of Martin’s procedure (synthesis of (+)-geissoschizine from D-tryptophan) [14]. All of these standard substances were identified using a NMR, MS, and infrared spectrometry method.

#### 3.1.3. Other Reagents

Pooled male and female human liver microsomes (pool of 50 donors, 20 mg protein/mL) were purchased from Gibco-BRL/Life Technologies, Inc. (Grand Island, NY, USA). Male Sprague-Dawley (SD) rat liver microsomes (20 mg protein/mL) and a nicotinamide adenine dinucleotide phosphate (NADPH)-regenerating system were purchased from BD Gentest (Woburn, MA, USA). Acetonitrile and methanol of LC/MS grade, formic acid, and ethyl acetate were purchased from Wako Pure Chemical Industries, Ltd. (Osaka, Japan). Water was purified using a pure water production system (MILLI-Q, Nihon Millipore; Tokyo, Japan).

### 3.2. Microsomal Incubations of GM and Sample Preparation

A stock solution of GM (2 mM) was prepared in 75% acetonitrile. The final concentration of acetonitrile in incubation was 0.75% (*v*/*v*). The rat and pooled human liver microsomes were carefully thawed on ice before the experiment. Microsomal incubations of GM were conducted by modification of the method described previously [8]. All incubations were performed at 37 °C in a water bath shaker. The rat or pooled human liver microsomal proteins (final 0.5 mg/mL) were added to a solution of GM (final 20 μM) in 100 mM potassium phosphate buffer (pH 7.4). The total incubation volume was 250 μL. After 3 min of preincubation at 37 °C, the incubation reactions were initiated by the addition of an NADPH regenerating system. To determine the incubation time, the microsomal incubations were performed at different time points (0, 5, 10, 20, 30, 40 and 60 min). The GM metabolite peaks sufficient for analysis were obtained at 60 min. After undergoing incubation for 60 min, the reactions were terminated by adding an equal volume of ethyl acetate. Control samples containing no NADPH or substrates were included. The stopped solution was mixed on a vortex for 3 min, and the supernatant was obtained by centrifugation at 16,000× *g* for 5 min at 4 °C (MRX-150; TOMY SEIKO Co., Ltd., Tokyo, Japan). The extraction was repeated twice. The corrected supernatant was dried by evaporation under gradual nitrogen flow at 40 °C. The dried residue was reconstituted in 200 μL of 0.2% formic acid containing 5% methanol, and the solution was mixed on a vortex. In total, 10 µL of the solution was injected into the LC/MS/MS system for analysis.

### 3.3. LC/MS/MS Analysis of GM and Its Metabolites

To elucidate and identify GM and its metabolites, the optimized LC/MS/MS method described previously was used [7]. An Agilent 1100 system (Agilent Technologies, Inc., Tokyo, Japan) comprising a vacuum degasser and a quaternary pump was used for solvent and sample delivery. An Ascentis Express RP-amide column (100 × 2.1 mm I.D., 2.7 μm particle size; Supelco Analytical, Inc., Tokyo, Japan) was used to separate each analyte for LC/MS/MS at 40 °C. The mobile phase comprised solution A (water: formic acid, 99.8%:0.2% *v*/*v*) and solution B (acetonitrile, 100% *v*/*v*), with a linear gradient of solution B (13%, 0 min; 15%, 12 min; 20%, 14 min; and 25%, 24 min; *v*/*v*) at a flow rate of 0.3 mL/min.

An API 4000 triple quadrupole mass spectrometer fitted with a Turbo IonSpray electrospray ionization instrument (Applied Biosystems Sciex, Tokyo, Japan) was used for MS and detection. Analyst ver.1.6.2 and Lightsight ver. 2.3 software (Applied Biosystems Sciex) were used for data acquisition and processing. MS data were obtained using information-dependent acquisition (IDA) experiments under positive-ion mode. In the IDA experiment, a selected reaction monitoring (SRM) scan was used as a survey scan, and if signals in the survey scan exceeded 200 cps, the enhanced product ion (EPI)-dependent scan was triggered. The EPI scan rate was 3000 amu/s, and a scan range of 10–500 amu was selected. The mass tolerance was set to 250 mmu, and the resolution of Q1 was set to unit. The dynamic exclusion criteria were set for 2 s after three occurrences. The collision energy (CE) was set at 30, 50, and 70 eV, with a CE spread of 15 eV. The declustering potential was set at 30 V, and a dynamic fill time function was used. The following additional parameters were used: ion spray voltage, 4000 V; temperature, 600 °C; ion source gas 1, 40 psi; ion source gas 2, 70 psi; curtain gas, 10 psi; collision-activated dissociation gas, 6 psi.

### 3.4. Identification of Demethyl GM

23-*O*-Demethyl GM and (±)-geissoschizine was dissolved in methanol to prepare stock solutions (100 μg/mL). The stock solutions were diluted with 0.2% formic acid containing 5% methanol to a final concentration of 100 ng/mL. Ten microliters of the solution was injected into the LC/MS/MS system for analysis. The LC/MS/MS conditions as described above and SRM (Q1, 353.2; Q3, 144.2) were used for the analysis of demethyl GM. The extracted-ion LC/MS/MS chromatograms of 23-*O*-demethyl GM and (±)-geissoschizine were compared with that of *m*/*z* 353.2 in the test samples.

## 4. Conclusions

In the present study, 13 GM metabolites from rat and human liver microsomes were elucidated and identified using a sensitive LC/MS/MS method, and their molecular structures were proposed on the basis of the characteristics of their precursor ions, product ions, and chromatographic retention times. There were no differences in the metabolites between the rat and human liver microsomes. The pathways of these metabolites may be mainly classified into five routes: demethylation, dehydrogenation, methylation, oxidation, and water-adduct metabolism. Among the 13 identified metabolites, there were two demethylation metabolites, one dehydrogenation metabolite, three methylation metabolites, three oxidation metabolites, two water-adduct metabolites, one di-demethylation metabolite, and one water-adduct metabolite followed by oxidation. The results from this study are important in understanding the metabolism of GM and related alkaloids of *Uncaria hook*, and may provide useful information on the pharmacokinetics and pharmacodynamics. This is the first report that describes the separation and identification of GM metabolites in human and rat liver microsomes.
